# Compositional Characterization of Ultrafine Composite Powder as a Novel Supplementary Cementitious Material

**DOI:** 10.3390/ma19153337

**Published:** 2026-08-05

**Authors:** Baoliang Li, Hongrui Shang, Liying Shi, Sahi Wail, Shouhua Liu, Yuanyang Chen, Binbin Huo

**Affiliations:** 1 Faculty of Architecture and Civil Engineering, Huai’an University, Huai’an 223001, China; lbljndx@126.com (B.L.); shang_hongrui@126.com (H.S.); sly18151607639@163.com (L.S.); liawisah@gmail.com (S.W.); 15312578936@163.com (Y.C.); 2 Jiangsu Key Laboratory of Construction Materials, School of Materials Science and Engineering, Southeast University, Nanjing 211189, China; huobinbin@cumt.edu.cn; 3 School of Mines, China University of Mining and Technology, Xuzhou 221116, China

**Keywords:** UCP, GBFS, ultrafine SCMs, composition, morphology, pores, particle size distribution

## Abstract

To investigate the application potential of ultrafine composite powder (UCP) as a novel supplementary cementitious material to replace ground granulated blast-furnace slag (GBFS) in cement-based materials and its underlying mechanism, this study first compared the activity differences between UCP and GBFS and their effects on mortar workability. Subsequently, multiple characterization techniques including XRF, XRD, TG/DTG, FTIR, mapping, SEM-EDS, and BET were employed to systematically examine the morphology, composition, particle size distribution, and pore structure characteristics of the two powders. Results show that UCP exhibits slightly higher 3 d and 28 d strength activity indices than GBFS, but contributes less to strength progression between 3 and 28 days. In terms of chemical composition, UCP contains lower combined CaO + MgO + Al_2_O_3_ content but significantly higher C and Fe levels and alkalinity than GBFS. Phase and microstructural analyses further reveal that UCP is predominantly composed of GBFS, fly ash (FA), steel slag, limestone powder, gypsum, superplasticizer, and alkaline activator, and is characterized as a mesoporous material with pores arising from fragmented FA, unburned carbon residues, and grinding-induced cracks. Quantitatively, the BET specific surface area, Blaine specific surface area, and total pore volume of UCP are 2.47, 1.59, and 3.31 times those of GBFS, respectively. Therefore, the early-age activity advantage of UCP is mainly attributed to the filling effect, the additional nucleation sites provided by its larger specific surface area, and the chemical activation induced by alkali and gypsum.

## 1. Introduction

Concrete is the most widely used man-made construction material in the world, with an annual production exceeding 3 billion tons [1]. However, this extensive use comes with significant environmental consequences: CO_2_ emissions from cement manufacturing account for approximately 8% of global man-made carbon emissions [2]; at the same time, the substantial consumption of limestone resources poses further challenges to sustainable development [3,4]. In the context of global efforts to reduce carbon emissions, the design of low-cement, green binder systems has emerged as a key research priority within concrete materials science. The partial replacement of cement with supplementary cementitious materials (SCMs) not only reduces cement consumption and associated carbon emissions but also enables the valorization of industrial solid wastes, aligning with the policy objectives of China’s “dual carbon” strategy [5]. Nevertheless, conventional SCMs, typified by fly ash (FA), generally exhibit relatively large particle sizes and low hydration activity and rates. These drawbacks limit both the early-age strength development of concrete and the maximum replacement level of SCMs in cementitious systems [6,7]. Previous studies [8,9,10] have demonstrated that reducing the particle size and increasing the specific surface area of SCMs can substantially enhance their reactivity in cement-based materials while simultaneously improving concrete durability [10]. Consequently, ultrafine SCMs have attracted growing interest from both the academic and engineering communities.

Ultrafine SCMs are generally defined as powders with an average particle size below 10 μm [11]. Their significantly reduced particle size endows them with a Blaine specific surface area exceeding 600 m^2^/kg, markedly higher than that of conventional SCMs (typically 300–500 m^2^/kg). This unique combination of high fineness, large specific surface area, and strong surface activity enables ultrafine SCMs to more effectively exert their filling, pozzolanic, and activation effects [12]. Based on their raw material sources, ultrafine SCMs can be categorized into three types: industrial solid-waste-derived (e.g., ultrafine ground granulated blast-furnace slag (GBFS) [13] and ultrafine FA [14]), natural mineral-derived (e.g., ultrafine limestone powder [15] and ultrafine metakaolin [16]), and synthetically produced (e.g., nano-SiO_2_ [17] and nano-CaCO_3_ [18]). Among these, industrial solid-waste-based ultrafine SCMs represent the most extensively studied and most diverse category of ultrafine powders to date.

Ultrafine SCMs can significantly enhance both the mechanical properties and durability of concrete through multiple mechanisms, including physical filling, optimization of particle size distribution, acceleration of nucleation of hydration products, and pozzolanic reaction. For instance, He et al. [19] demonstrated that replacing 90% of cement with ultrafine GBFS still effectively improves the mechanical performance of concrete, indicating that the reactivity of GBFS after ultrafine grinding is comparable to that of cement, thus enabling a substantial increase in the resource utilization rate of industrial solid wastes. Similarly, Jaturapitakkul et al. [20] observed that concrete containing 15–35% ultrafine FA developed compressive strengths at 60 days and later ages that were equal to or greater than those of silica-fume-blended concrete. In a related study, Han et al. [21] found that ultrafine FA, acting through both filler effects and pozzolanic reactivity, refines the internal pore structure, significantly decreasing the volume fraction of pores larger than 100 nm. Furthermore, Zong et al. [22] reported that the addition of 20–30% ultrafine SCMs not only enhanced the 7-day compressive and flexural strengths of pavement concrete, but also boosted its freeze–thaw resistance by 1.6 times and its resistance to chloride ion penetration by nearly 4.0 times. Beyond these performance gains, ultrafine SCMs also contribute to improved sulfate resistance, primarily by consuming calcium hydroxide (CH) produced during cement hydration, reducing the effective C_3_A content, and further densifying the matrix microstructure [23].

The effect of ultrafine SCMs on the carbonation resistance of concrete is governed by two competing mechanisms. On one hand, the densification of the matrix pore structure impedes CO_2_ diffusion and internal moisture transport, thereby retarding the carbonation process. On the other hand, the pozzolanic reaction of ultrafine SCMs consumes substantial amounts of CH generated from cement hydration, which in turn accelerates the carbonation reaction rate. Consequently, the net impact of ultrafine SCMs on concrete carbonation behavior is determined by the interplay of dosage, water-to-binder ratio, and curing conditions [24,25]. Existing studies indicate that a replacement level of 10–20% effectively enhances carbonation resistance, whereas dosages exceeding 30% lead to a pronounced deterioration in carbonation performance [24,25].

However, the ultrafine grinding of SCMs introduces several practical challenges. It significantly increases the water demand, yield stress, and plastic viscosity of cementitious pastes [26], while also exacerbating autogenous shrinkage deformation [13]. More importantly, the energy-intensive nature of ultrafine grinding drives up both production costs and final market prices, posing a significant barrier to large-scale field deployment. To resolve the inherent trade-off between cost and mechanical performance, the preparation of ultrafine SCMs through multi-component blending represents a practical and effective approach. For instance, binary ultrafine SCMs can be produced by combining ultrafine GBFS with ultrafine FA, steel slag powder, or limestone powder. Compared with their single-component counterparts (e.g., ultrafine GBFS alone), these composite blends offer superior capabilities in regulating cement hydration, refining and densifying the matrix pore structure, and reducing raw material costs [27,28,29]. Therefore, they hold considerable promise for widespread application in civil engineering.

At present, a new category of composite ultrafine SCM—referred to as “ultrafine composite powder (UCP)”—has emerged in the building materials market in China. This product offers comprehensive performance comparable to that of conventional GBFS, yet at a lower cost, and has been widely adopted in engineering practice as a substitute for GBFS. However, the chemical and mineral composition of UCP, the mechanism underlying its potential as a GBFS replacement in concrete, and its effects on concrete strength and durability remain largely unclear. To fill this knowledge gap, the present study employs conventional GBFS as a reference and utilizes a comprehensive suite of microstructural characterization techniques—including XRF, XRD, TG/DTG, FTIR, elemental mapping, SEM–EDS, and BET—to systematically analyze the phase composition and microstructural characteristics of this commercial UCP, with the aim of elucidating its action mechanism in concrete from a compositional perspective. The findings are expected to provide a theoretical basis and technical support for the large-scale engineering application of this novel ultrafine SCM.

## 2. Materials and Methods

### 2.1. Materials

P·O42.5 Ordinary Portland Cement (OPC) meeting Chinese National Standard GB 175-2023 [30] was supplied by Huai’an Conch Cement Co., Ltd. (Huai’an, China). Its specific surface area was 358 m^2^/kg.

The Type S95 ground granulated blast-furnace slag (GBFS) and ultrafine composite powder (UCP) used in this study are both commercially available products, supplied by Jiangsu Jiaru New Building Materials Co., Ltd. (Huai’an, China).

Standard sand with a fineness modulus of 2.75, conforming to GB/T 17671-2021 [31], was used to prepare mortar and to conduct the fluidity test.

Distilled water was adopted as mixing water in this study to prepare cement paste, cement mortar, and the suspension required for pH measurement.

### 2.2. Sample Preparation and Test Methods

#### 2.2.1. Sample Preparation

The reactivity of GBFS is typically determined by replacing 50% of Portland cement with an equal mass of GBFS to prepare cement mortars. The compressive strength is measured at 28 days, and the reactivity index is expressed as the percentage ratio of this strength to that of the corresponding plain cement mortar tested under identical conditions. Therefore, to assess the effect of UCP on the basic properties of cement-based materials, pastes and mortars were prepared with 50% cement replacement by either GBFS or UCP under the same experimental setup. The setting time, flowability, and compressive strength were systematically compared between the two series. The water-to-binder ratios were 0.3 for pastes and 0.5 for mortars, while the binder-to-sand ratio in mortar was maintained at 1:3 by mass.

Mechanical properties were evaluated on mortar prism specimens with dimensions of 40 mm × 40 mm × 160 mm, prepared and tested according to the Chinese standard GB/T 17671-2021 [31], which covers both compressive and flexural strength measurements.

For pH measurement, GBFS and UCP were separately suspended in distilled water at mass ratios of 1:2, 1:5, 1:8, and 1:10. After vigorous stirring with a glass rod for 1–2 min, the suspensions were left to rest for 30 min, and the pH values were recorded using a calibrated pH electrode immersed in the suspensions.

#### 2.2.2. Test Methods

To facilitate a clear and intuitive overview of the key design factors and operational parameters adopted in the various tests of this study, the relevant information is summarized in Table 1.

As shown in Table 1, the setting time of cement paste incorporating UCP and GBFS was measured using a Vicat apparatus (Hebei Tongli Instrument Equipment Co., Ltd., Cangzhou, China) at a water-to-cement ratio of 0.3, in accordance with the Chinese national standard GB/T 1346-2024 [32]. Cement mortar was prepared with a binder-to-standard sand-to-water mass ratio of 450:1350:225, and its fluidity was determined by the flow table method following GB/T 2419-2005 [33].

The raw materials were comprehensively characterized using multiple analytical techniques. The Blaine specific surface areas of GBFS and UCP were measured with a Blaine air-permeability apparatus (Beijing Zhongjiaojianyi Technology Co., Ltd., Beijing, China), based on the time required for air to pass through a compacted powder bed at constant pressure.

The particle size distribution was determined using a Microtrac S3500 laser diffraction particle size analyzer (Microtrac Inc., York, PA, USA) via laser scattering; prior to measurement, 50–100 mg of sample was dispersed in anhydrous ethanol and ultrasonicated for 2 min, and each sample was analyzed in triplicate.

The chemical composition was analyzed by X-ray fluorescence spectroscopy (XRF, Thermo Fisher Scientific, Waltham, MA, USA), while the phase composition was characterized by a D8-Discover X-ray diffractometer (XRD, Bruker, Karlsruhe, Germany) operating at a scan rate of 3°/min.

Particle morphology, phase distribution, and elemental composition were examined using a Sirion field-emission scanning electron microscope (SEM, FEI Company, Hillsboro, OR, USA) equipped with an energy-dispersive X-ray spectrometer (EDX), with elemental mapping simultaneously performed to obtain spatial distribution information.

Thermal behavior and phase transformations were evaluated by thermogravimetric/differential thermogravimetric analysis (TG/DTG) using a STA449 F3 synchronous thermal analyzer (Selb, Germany), under a N_2_ atmosphere at a heating rate of 10 °C/min from 60 °C to 1000 °C.

The differences in functional groups and chemical bonds between GBFS and UCP were further characterized by Fourier transform infrared spectroscopy (FT-IR, DEAUPOS SCIENTIFIC, Berlin, Germany), with spectra recorded at a resolution of 4 cm^−1^ over a wavenumber range of 500–4000 cm^−1^ (32 cumulative scans).

Additionally, the pore structure and specific surface area were characterized using a fully automated gas adsorption analyzer (ASIQM0001-5, Quantachrome Instruments, Boynton Beach, FL, USA); pore characteristics were derived from adsorption–desorption isotherm analysis, and the measurements were carried out at 77 K using N_2_ as the adsorbate over a relative pressure (P/P_0_) range of 0.05–0.995.

## 3. Results and Discussion

### 3.1. Setting Time and Fluidity

To identify the distinctive features of UCP as an SCM relative to the conventional counterpart GBFS, this study first investigated the effects of UCP on the setting time of cement paste and the fluidity of mortar. Both materials were tested at a 50% cement replacement level, and the results are summarized in Table 2. Although both UCP and GBFS significantly delayed cement setting, UCP exhibited a slightly shorter retardation in both initial and final setting times compared to GBFS, suggesting its superior early-age activity.

Furthermore, the fluidity of plain cement mortar was 205 mm, and it increased with the incorporation of either UCP or GBFS. Although SCM particles are generally finer than cement grains, their beneficial effect on concrete workability can be attributed to the following aspects:

(1) Particle filling effect. The fine SCM particles fill the voids between cement grains, thereby increasing the packing density of the cementitious system and reducing internal frictional resistance, which facilitates the flow of paste under self-weight or slight vibration [34].
(2) Particle morphology. Fly ash-based SCMs typically exhibit high sphericity and a smooth surface, acting like “ball bearings” that effectively reduce shear resistance between particles [35]. In contrast, cement particles are mostly irregular and angular, with strong interlocking effects that increase flow resistance.
(3) Modulation of hydration rate. The early-age hydration activity of GBFS and FA is lower than that of cement; this relative “inertness” delays the rapid formation of hydration products such as C−S−H gels and ettringite, preventing a sharp increase in paste viscosity within a short period and thus extending the retention time of workability [36].
(4) Reduction in water demand. Under equal mass replacement conditions, GBFS and FA typically have slightly lower densities than cement and do not participate in intense early hydration reactions, resulting in a lower water demand for the neat paste. At the same water-to-binder ratio, this releases more “free water,” which is conducive to improving paste fluidity.
(5) Water-reducing effect of superplasticizer. Although UCP possesses a high specific surface area, its negative impact on mortar flowability is relatively limited. This may be attributed to the incorporation of water-reducing components during its production process. However, the addition of such components inevitably increases the production cost of UCP.

However, the mortar fluidity of U50 was slightly lower than that of G50, which may be attributable to the finer particle size and larger specific surface area of UCP, leading to a relatively higher water demand.

### 3.2. Mechanical Properties of Mortar

**Figure 1:**
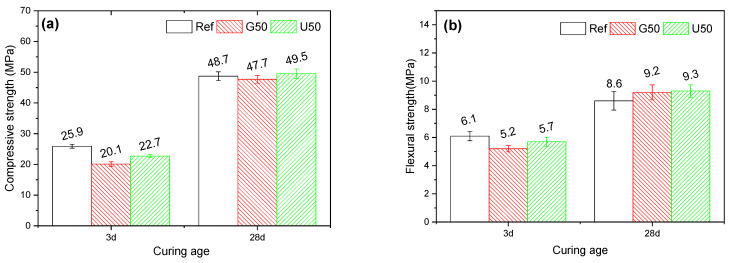
Strength of G50 an U50 mortars: (**a**) compressive strength, (**b**) flexural strength.

To evaluate the activity of UCP, mortars were prepared with 50% cement replacement by either UCP or GBFS, and their mechanical properties at 3 d and 28 d were tested, with the results presented in Figure 1. It can be observed that UCP exhibits higher early-age activity compared to GBFS. Specifically, at 3 d, the compressive strength activity indices of GBFS and UCP were 77.6% and 87.6%, respectively, while at 28 d, they reached 97.9% and 101.6%. The flexural strength activity indices of GBFS and UCP at 3 d were 85.2% and 93.4%, respectively, and increased to 107.0% and 108.1% at 28 d. Although UCP exhibited higher activity indices at both 3 d and 28 d, the increment in activity indices from 3 d to 28 d for UCP was lower than that for GBFS, for both compressive and flexural strengths, indicating that the activity advantage of UCP over GBFS is primarily manifested at early ages.

In addition, it was found that the flexural strength activity indices of both G50 (GBFS blended mortar) and U50 (UCP blended mortar) were higher than their corresponding compressive strength activity indices, at both 3 d and 28 d. This can be primarily attributed to the following factors: (1) Filling effect. By filling the voids between cement particles, GBFS and UCP significantly reduce porosity and refine the pore size distribution. This densification increases the resistance to microcrack initiation and propagation in mortar under external loading, thereby contributing more effectively to the enhancement of flexural strength [34]. (2) Improvement of the interfacial transition zone. GBFS and UCP can react with calcium hydroxide (CH) generated during cement hydration, consuming CH and reducing its crystal size while simultaneously forming additional C−S−H gel. This reaction improves the interfacial transition zone (ITZ) and enhances the overall microstructure [37]. It should be noted that GBFS is not generally considered a pozzolanic material. Owing to its high CaO content, its consumption of CH in cement concrete is less pronounced than that of typical pozzolanic materials such as FA and silica fume. (3) Hydration kinetics: The hydration rate of GBFS and UCP is relatively slower than that of cement. Consequently, the C−S−H gel formed in the early stage within mortars containing GBFS or UCP is more uniformly dispersed and exhibits a denser microstructure, facilitating the development of a continuous network skeleton that enhances the toughness of the concrete [38].

### 3.3. Appearance of UCP Chemical Composition

**Figure 2:**
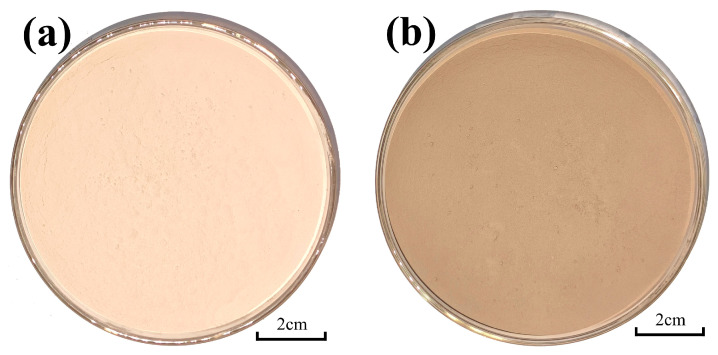
Appearance of UCP: (**a**) GBFS, (**b**) UCP.

To investigate the reasons for the higher early-age activity of UCP, the appearances of GBFS and UCP were first compared, with the results shown in Figure 2. GBFS (Figure 2a) exhibited a relatively light milky white or pale yellow color, while UCP (Figure 2b) appeared darker, predominantly dark gray. Upon gentle vibration of the Petri dishes containing the powders, it was observed that GBFS particles were more prone to forming discontinuities (i.e., cracking or segregation) between them, whereas UCP displayed better flowability. This may be attributed to its more favorable particle size distribution. A well-graded particle size distribution helps reduce frictional resistance between particles, thereby enabling UCP to flow more readily under self-weight or slight vibration.

### 3.4. Chemical Composition

Table 3 summarizes the chemical compositions of GBFS and UCP. The two powders exhibit generally similar oxide profiles, though with several notable distinctions. The CaO content of UCP was lower than that of GBFS, whereas their Al_2_O_3_ and SiO_2_ contents were relatively comparable. As a result, the Ca/Si molar ratio of UCP (1.14) was lower than that of GBFS (1.51). Under equal-mass cement replacement, UCP thus exerts a stronger dilution effect on the overall Ca/Si ratio of the paste, making it more effective in reducing the Ca/Si ratio of the C−S−H gel in hydration products. Additionally, the MgO content of UCP was slightly lower than that of GBFS. Since CaO, Al_2_O_3_, and MgO are generally considered to enhance hydraulic activity [39], the characteristic coefficients of the two powders were calculated using the formula (%CaO + %MgO + %Al_2_O_3_)/(%SiO_2_ + %TiO_2_) [40], yielding values of 2.20 for GBFS and 1.84 for UCP. Based on this calculation, it was inferred that UCP would exhibit lower cementitious activity than GBFS. However, this inference is contradicted by the experimental results shown in Figure 1, indicating that this empirical formula is not applicable for evaluating the hydration activity of UCP.

The SO_3_ content in UCP was significantly higher than that in GBFS. In supplementary cementitious materials, SO_3_ generally exists in the form of gypsum, which primarily acts as a set regulator and chemical activator [41]; moreover, an appropriate amount of gypsum is beneficial for optimizing the overall performance of the paste [42]. Therefore, the higher SO_3_ content in UCP contributes not only to its strength development but also to the stability of workability. In addition, the contents of Fe_2_O_3_ and MnO in UCP were higher than those in GBFS, which is most likely the main reason for the darker color of UCP [43,44,45].

**Figure 3:**
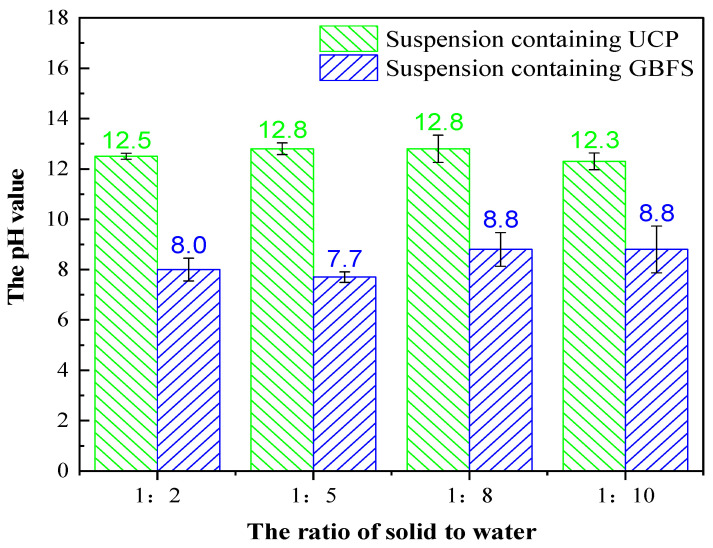
The pH values of suspensions containing GBFS and UCP under different solid–liquid ratios.

In addition, the Na_2_O content of UCP was found to be higher than that of GBFS, which is most likely attributable to the introduction of alkaline activators containing Na_2_O during the preparation process to enhance the cementitious activity of UCP. To verify this hypothesis, UCP and GBFS were individually stirred in distilled water at solid-to-liquid ratios of 1:2, 1:5, 1:8, and 1:10. After stirring for 30 min, the pH values of the suspensions were measured, and the results are presented in Figure 3. Regardless of the solid-to-liquid ratio, the UCP–water mixtures consistently exhibited higher pH values than their GBFS counterparts, which may also account for the higher reactivity observed for UCP.

### 3.5. Phase Composition

#### 3.5.1. XRD

**Figure 4:**
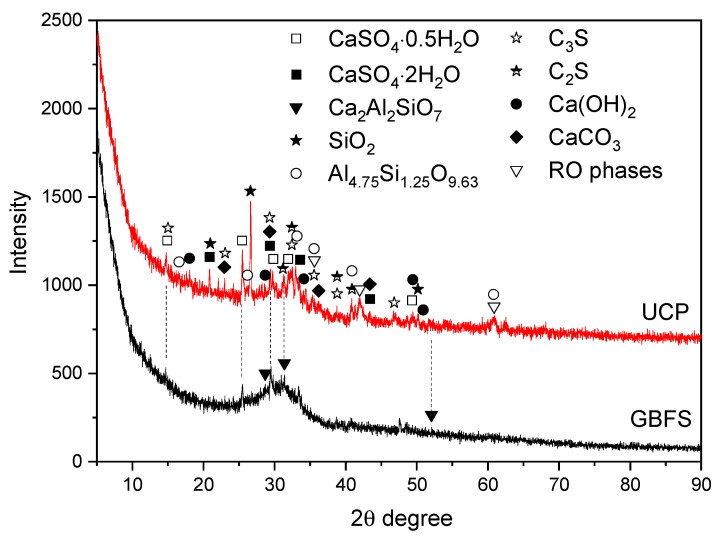
X-Ray diffraction pattern of GBFS and UCP.

The XRD patterns of GBFS and UCP are shown in Figure 4. GBFS predominantly exhibited an amorphous phase as the main phase, along with minor amounts of gehlenite (Ca_2_Al_2_SiO_7_) and calcium sulfate hemihydrate (CaSO_4_·0.5H_2_O). Gehlenite is a stable crystalline phase formed during the high-temperature melting and rapid cooling of GBFS [46]. Gypsum, commonly used as an activator in GBFS, undergoes dehydration to hemihydrate during the grinding process due to the substantial heat released.

In contrast, the phase assemblage of UCP was considerably more complex. In addition to the GBFS-derived phases mentioned above, UCP contained gypsum (CaSO_4_·2H_2_O), quartz (SiO_2_), mullite (Al_4_._75_Si_1_._25_O_9_._63_), tricalcium silicate (C_3_S), dicalcium silicate (C_2_S), portlandite (Ca(OH)_2_), calcite (CaCO_3_), and RO phases. Among these, quartz and mullite are characteristic phases of FA [37]; C_3_S, C_2_S, Ca(OH)_2_, and RO phases are typical constituents of steel slag [47]; and calcite is the main mineral component of limestone powder. Based on the above analysis, UCP can be preliminarily identified as a composite material composed of GBFS, FA, steel slag powder, limestone powder, and gypsum.

#### 3.5.2. TG-DTG

**Figure 5:**
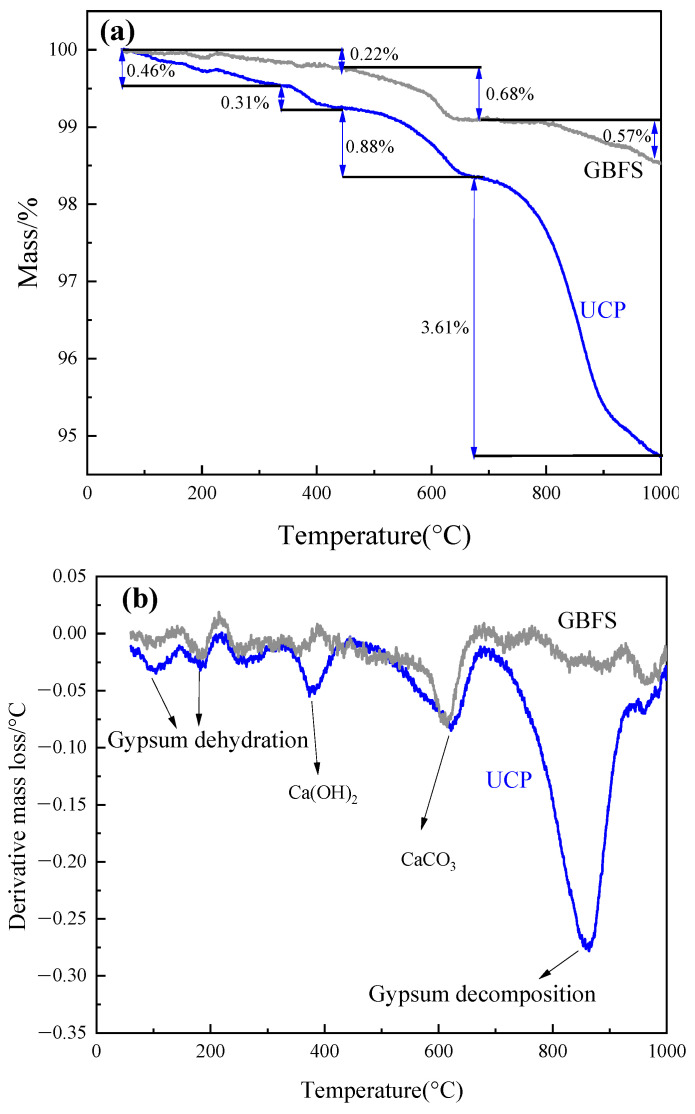
TG (**a**) and DTG (**b**) curves for GBFS and UCP.

The TG/DTG curves of GBFS and UCP in the temperature range of 60–1000 °C are presented in Figure 5. In thermal analysis studies of cement-based materials, mass loss in different temperature intervals typically corresponds to the decomposition of specific phases. Generally, the mass loss from room temperature to 200 °C is mainly attributed to the evaporation of free water adsorbed on particle surfaces and the initial dehydration of certain hydrates. For instance, ettringite decomposes above 80 °C, while gypsum dehydrates to hemihydrate at 107–170 °C [48]. The mass loss in the 400–500 °C range is characteristic of the dehydroxylation of calcium hydroxide (Ca(OH)_2_) [49], whereas that in the 600–750 °C range primarily corresponds to the decomposition of carbonates such as CaCO_3_ [50]. Above 800 °C, the mass loss is generally associated with the decomposition of sulfates such as gypsum [51].

As shown in Figure 5, GBFS exhibited a very small mass loss, with a total weight loss of only 1.48%. The thermal decomposition of GBFS mainly occurred in two stages: the removal of a very small amount of physically adsorbed water at low temperatures, and the decomposition of carbonates at around 600 °C. In addition, GBFS contained a small amount of gypsum, which dehydrates below 200 °C and gradually decomposes above 800 °C.

In contrast, UCP showed a significantly larger mass loss, with a total weight loss of 5.48%. Its decomposition could be divided into three main stages. First, in addition to the removal of physically adsorbed water, an obvious weight-loss peak appeared at around 400 °C, which is mainly attributed to the decomposition of Ca(OH)_2_. This calcium hydroxide is primarily derived from the hydration of cement clinker minerals present in steel slag, as well as the conversion of free CaO (*f*-CaO) upon absorbing moisture. Second, the weight-loss peak at approximately 600 °C was mainly caused by carbonate decomposition. Finally, the sharp decline in the curve above 800 °C is attributable to the high-temperature decomposition of gypsum, which serves as an activator in UCP.

#### 3.5.3. FTIR

**Figure 6:**
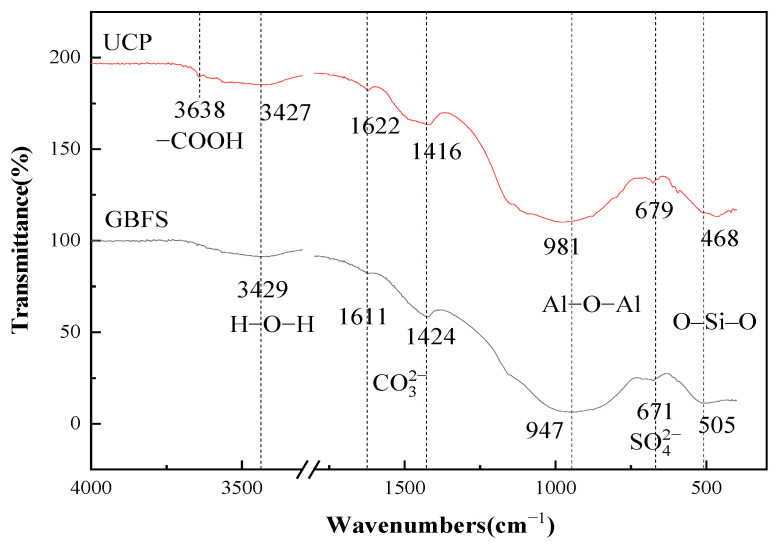
FTIR curves for GBFS and UCP.

The FTIR spectra of GBFS and UCP are shown in Figure 6. Fourier transform infrared spectroscopy can reveal the evolution of functional groups in cement-based materials from the perspective of molecular vibrational energy levels. In cement-based materials, the absorption bands in the range of 3000–4000 cm^−1^ are generally attributed to the stretching vibrations of hydroxyl groups (–OH), free water, and chemically bound water [40]; the bands between 1400 and 1650 cm^−1^ correspond to the bending vibrations of carbonate (CO_3_^2−^) and water molecules [52]; and the fingerprint region from 400 to 1200 cm^−1^ mainly reflects the structural characteristics of the silicon (aluminum) oxygen tetrahedral skeleton (Si–O–Si or Si–O–Al) as well as sulfate (SO_4_^2−^) groups [53]. Overall, the peak shapes of GBFS and UCP were basically identical, indicating that UCP is largely composed of GBFS.

The infrared spectrum of GBFS exhibited a relatively smooth profile. A weak free-water adsorption band was observed at 3429 cm^−1^ and 1611 cm^−1^, and a prominent broad absorption peak was observed at 947 cm^−1^, which corresponds to the asymmetric stretching vibration of the SiO_4_ tetrahedra in GBFS. In addition, a small peak was detected at 1424 cm^−1^, indicating the presence of minor carbonate species in GBFS, a finding that is consistent with the analysis presented in Figure 5.

In contrast, the infrared spectrum of UCP exhibited a sharp absorption peak at 3638 cm^−1^. This feature is not only associated with the presence of CH in UCP, but may also be related to the existence of carboxyl (–COOH) and hydroxyl (–OH) groups, which is presumably attributable to the possible incorporation of a water-reducing agent in UCP. Meanwhile, the characteristic band corresponding to the silicate skeleton vibration shifted from 947 cm^−1^ (as observed in GBFS) to a higher wavenumber of 981 cm^−1^, and the peak became markedly sharper. This shift indicates a higher degree of polymerization of the SiO_4_ tetrahedra and a lower overall Ca/Si ratio in UCP [54], which is consistent with the XRF analysis. It should be noted, however, that the characteristic bands corresponding to the silicate skeleton vibration are relatively broad in the FTIR spectra of both GBFS and UCP. The observed peak shift from 947 cm^−1^ to 981 cm^−1^ may therefore be influenced by overlapping contributions from the amorphous phases in GBFS, the vitreous phases in FA, residual crystalline quartz, and the newly formed calcium-silicate-hydrate (C−S−H) gel. In addition, an enhanced bending vibration of SO_4_^2−^ was observed at around 679 cm^−1^, reflecting a higher sulfate content in UCP than in GBFS.

### 3.6. Morphology and Composition Under SEM

#### 3.6.1. SEM and Elemental Mapping

**Figure 7:**
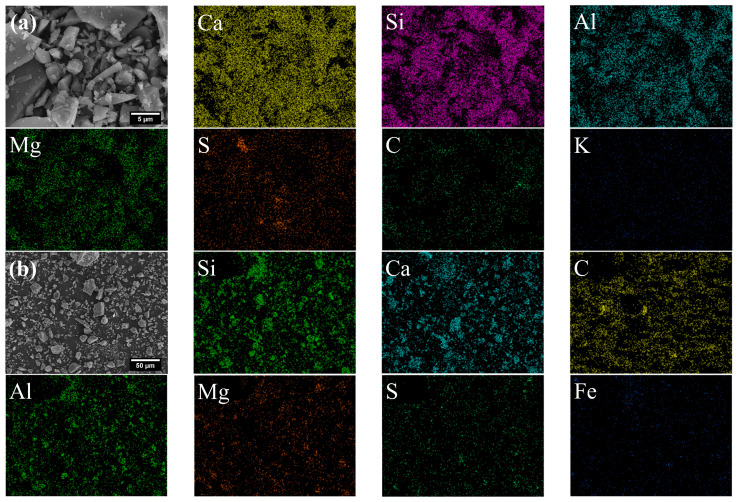
SEM and elemental mapping of (**a**) GBFS and (**b**) UCP.

Figure 7 presents the SEM images and corresponding elemental mapping results of GBFS and UCP. In terms of morphology, GBFS particles were predominantly irregularly blocky or angular (Figure 7a), with a wide particle size distribution, and their main elemental composition included Ca, Si, Al, Mg, S, C, and K. In contrast, UCP exhibited a smaller particle size, a narrower size distribution, and more diverse morphologies; apart from blocky particles, spherical particles were also clearly observable (Figure 7b). This finer particle size and the presence of small spherical shapes endow UCP with superior filling performance. Regarding elemental composition, UCP mainly consists of Si, Ca, C, Al, Mg, S, and Fe, with relatively higher contents of C and Fe compared to GBFS. In addition to the elements Fe and Mn, the higher C content also contributes to the darker color appearance of UCP.

#### 3.6.2. SEM-EDX

**Figure 8:**
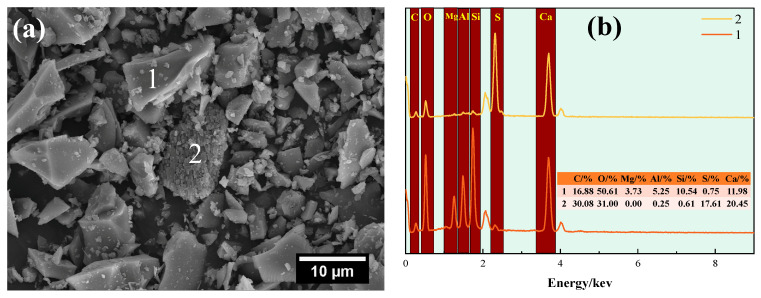
The morphology and composition of GBFS under SEM, (**a**) morphology of GBFS, (**b**) EDX results of particle 1 (original GBFS) and particle 2 (gypsum).

**Figure 9:**
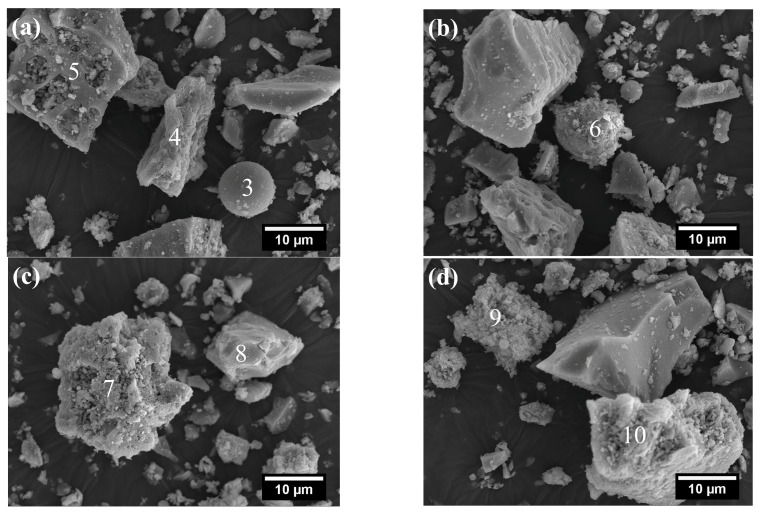
The SEM-observed morphology of various constituent phases within UCP, (**a**) FA and unburned C particles, (**b**) C_2_S, (**c**) RO phase and GBFS particles, (**d**) calcium carbonate and gypsum particles. Note: Particles 3–10 correspond to the following phases: 3, FA; 4, C; 5, crushed FA; 6, C_2_S in steel slag; 7, RO phase in steel slag; 8, GBFS; 9, CaCO_3_; and 10, gypsum.

**Figure 10:**
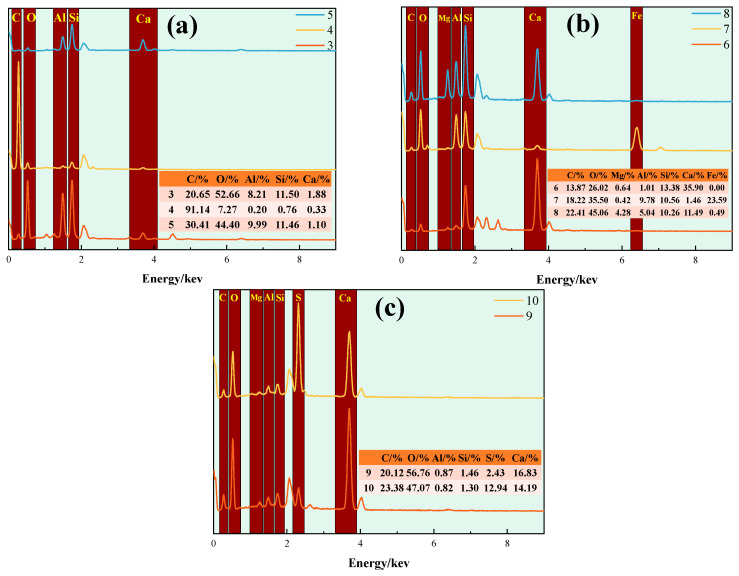
EDX results of particles 3~10 from Figure 9, (**a**) particles 3~5, (**b**) particles 6~8, (**c**) particles 9~10.

To gain a clearer understanding of the phase compositions of UCP and GBFS, selected regions of GBFS and UCP were magnified, and the phases with distinctive morphologies were identified, with the results shown in Figure 8, Figure 9 and Figure 10.

As presented in Figure 8, GBFS mainly consisted of two types of particles. One type was irregularly shaped massive particles, as exemplified by particle 1 in Figure 8, which were mainly composed of Ca, Si, Al, and Mg. Based on this composition, these particles are identified as the original GBFS particles and constitute the major component of GBFS. In addition, a porous particle (particle 2) was also observed in Figure 8, with its composition dominated by Ca, S, and O. Accordingly, this particle is identified as gypsum, which is consistent with the analysis in Figure 4.

Particle 3 in Figure 9a exhibited a spherical morphology, with fine particles attached to its surface. The EDX analysis in Figure 10a revealed that this particle was mainly composed of O, C, Si, and Al, with only a very low content of Ca. Based on its morphology and chemical composition, this particle is identified as a FA particle. The presence of spherical FA particles in UCP contributes significantly to improving the flowability of concrete.

Particle 4 in Figure 9a exhibited a loose and porous morphology, with its carbon content reaching as high as 91.14% (Figure 10a), suggesting that this particle is likely unburned carbonaceous residue in FA. Such porous carbonaceous particles possess strong water absorption characteristics, which not only adversely affect the workability of concrete but also significantly increase the loss on ignition of UCP.

Particle 5 in Figure 9a exhibited a fragmented porous structure resembling a “lotus root” shape. In addition to carbon, its chemical composition was dominated by O, Si, and Al. Based on literature analysis, these particles are identified as fragmented FA particles generated during the ultrafine grinding process [37]. Their presence not only raises the water demand and lowers the slump of concrete, but also exerts an influence on the viscosity of the fresh mixture [55].

Particle 6 in Figure 9b exhibited a spheroidal morphology. In addition to carbon, its chemical composition was mainly Ca, O, and Si, with a Ca/Si ratio intermediate between that of C_2_S and C_3_S. Based on its morphology and composition [56], this particle is identified as C_2_S in steel slag, which is consistent with the XRD analysis in Figure 4. The presence of C_2_S not only improves the cementitious property of UCP, but also enhances its alkalinity.

In Figure 9c, particle 7 exhibited a porous morphology and a relatively large particle size, approaching 20 μm. Combined with the compositional data of this particle shown in Figure 10b, it was found that the particle was mainly composed of O and Fe, along with minor amounts of Si, Al, and other elements. On this basis, it is preliminarily inferred that the particle is likely the RO phase in steel slag [57]; its large particle size also indicates that it is not readily ground during pulverization.

In Figure 9c, particle 8 exhibited a blocky morphology with a particle size of approximately 10 μm, and it was identified as a major phase in the UCP. Combined with the compositional analysis of this particle in Figure 10b, it was revealed that, apart from C and O, the particle was mainly composed of Ca, Si, Al, and Mg. Its morphology and composition were found to be closely comparable to those of particle 1 in Figure 8a, suggesting that it is most likely a GBFS particle.

In Figure 9d, particle 9 was observed to be an agglomerate composed of numerous fine particles. EDX analysis (Figure 10c) revealed that the particle was primarily composed of O, C, and Ca, allowing it to be identified as CaCO_3_. This CaCO_3_ is likely derived from limestone powder or steel slag, and it serves primarily as a filler in concrete.

In Figure 9d, particle 10 was found to be mainly composed of O, C, Ca, and S (Figure 10c). On the basis of its composition, it was identified as gypsum (CaSO_4_·2H_2_O), which is consistent with the aforementioned XRD analysis. Gypsum plays multiple roles in the system: it acts as a retarder to regulate the setting time, thereby ensuring a sufficient workable window for construction; simultaneously, it serves as a chemical activator to induce the hydration of SCMs, which is beneficial for the synchronous enhancement of both later-age strength and durability of concrete [42].

In summary, the UCP is mainly composed of GBFS, FA, steel slag powder, limestone powder, and gypsum. Among these components, GBFS and steel slag powder serve as the primary sources of reactivity. Although spherical FA particles can improve the fluidity of the cementitious system, the presence of irregular porous FA particles and unburned carbon particles tends to increase the water demand of concrete. Limestone powder, which is predominantly composed of calcium carbonate, mainly functions as a filler [58]. Gypsum is incorporated primarily to activate the reactivity of GBFS, FA, and steel slag powder.

### 3.7. Particle Size and Fineness Analysis

**Figure 11:**
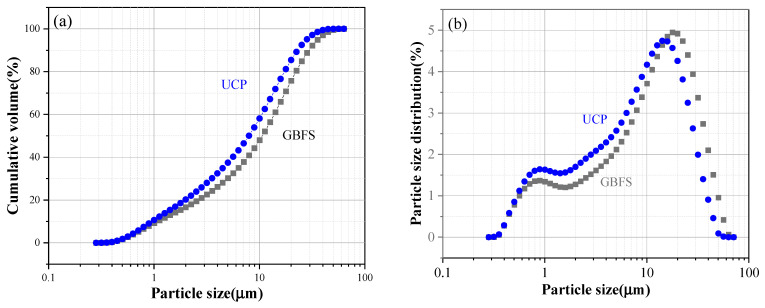
Particle size distribution of GBFS and UCP, (**a**) Cumulative volume, (**b**) Particle size distribution.

The particle size distribution curves of GBFS and UCP are shown in Figure 11, and the corresponding key parameters—including maximum particle size, mean particle size, most probable particle size, D10, D50, D90, and specific surface area—are summarized in Table 4.

The particle size distribution of GBFS exhibited a distinct bimodal pattern, with a primary peak located in the range of 10–20 μm and a secondary peak appearing around 1 μm. The key size parameters of GBFS were determined as follows: the median particle size (D50) was 10.6 μm, D90 was 29.4 μm, and the Blaine specific surface area was 388 m^2^/kg. Overall, the GBFS particles were relatively coarse, with a notably high proportion of coarse fractions.

Similarly to GBFS, the particle size distribution of UCP also exhibited a bimodal pattern; however, the entire distribution curve shifted toward the finer side. The median particle size (D50) of UCP was 7.9 μm, which was approximately 25.5% lower than that of GBFS. The D90 decreased from 29.4 μm to 23.1 μm, and the residue on the 45 μm sieve was nearly zero. The specific surface area of UCP reached 618 m^2^/kg, representing an increase of about 59.3% compared to that of GBFS.

In comparison with GBFS, the UCP particles were significantly finer, with a greatly reduced proportion of coarse fractions and a narrower overall size distribution. Such fine particles can provide abundant nucleation sites for the hydration products of cement-based materials, which favors the densification of the microstructure at later ages.

### 3.8. Pore Structure

#### 3.8.1. The N_2_ Adsorption/Desorption Isotherms and Pore Structure Characteristics of UCP

**Figure 12:**
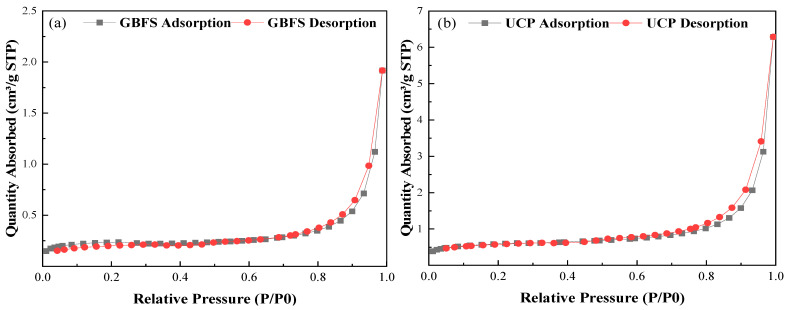
N_2_ adsorption–desorption isotherms of (**a**) GBFS and (**b**) UCP.

The N_2_ adsorption/desorption isotherms of GBFS and UCP are presented in Figure 12. The results showed that the adsorption isotherms of both GBFS and UCP conformed to the Type IV isotherm characteristics according to the IUPAC classification [59], and distinct hysteresis loops were observed at relatively high relative pressures (P/P_0_ > 0.8), indicating that both GBFS and UCP are mesoporous materials.

As shown in Figure 12a, the N_2_ adsorption capacity of GBFS was relatively low, with a maximum adsorption value of 2.0 cm^3^/g. In contrast, the adsorption performance of UCP was significantly enhanced, as presented in Figure 12b, where the maximum adsorption capacity reached 6.3 cm^3^/g—more than three times that of GBFS. At relative pressures below 0.4 (P/P_0_ < 0.4), the adsorption and desorption curves of both GBFS and UCP were very close to each other and exhibited a relatively small slope, suggesting the presence of a small number of cylindrical pores with one end closed (e.g., broken FA particles in Figure 9a), parallel-plate slit-shaped pores (e.g., microcracks generated during grinding), or ink-bottle pores (corresponding to unburned carbon particles in UCP in Figure 9a) within the pore size range of <2 nm.

According to the shape of the hysteresis loops, the isotherms of both GBFS and UCP were closer to the H3 type [60]. This type of loop is typically associated with slit-shaped pores formed by the stacking of plate-like or layered particles. In the case of GBFS, such pore structures are mainly attributed to microcracks in the amorphous phase induced by the rapid quenching process. For UCP, additionally, they arise from thermal cracks developed during the cooling of various steel slag phases, as well as numerous physical defects and microcracks generated during the ultrafine grinding process. These microcracks are more favorable for water penetration at the early stage of hydration—though they also inevitably increase the water demand.

A comparison of the pore structures between GBFS and UCP revealed that UCP possessed a more developed pore network and a larger effective reaction interface than GBFS. This optimization of the microstructural architecture provides a solid physical foundation for UCP to exert excellent physical filling effects and interface-induced hydration promotion in cement-based materials.

#### 3.8.2. Pore Size Distribution

**Figure 13:**
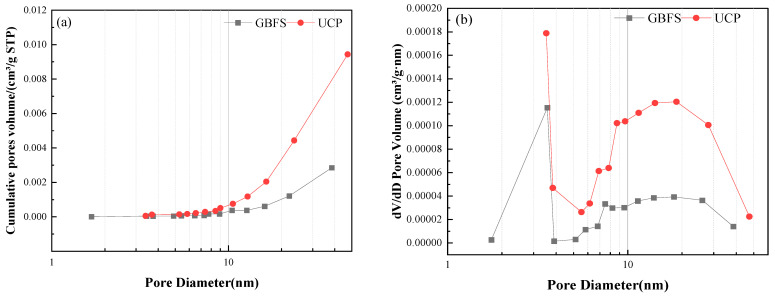
Cumulative pore volume (**a**) and pore size distribution plots (**b**) of GBFS and UCP.

According to the N_2_ adsorption process, pores in porous materials can be classified into micropores (<2 nm), mesopores (2–50 nm), and macropores (>50 nm) [59]. The cumulative pore volume and pore size distributions of GBFS and UCP are presented in Figure 13. As shown in the cumulative pore volume plot (Figure 13a), UCP exhibited a significantly higher cumulative pore volume than GBFS, approximately three times that of the latter. The pore sizes of both GBFS and UCP were below 50 nm, indicating that both materials were predominantly composed of mesopores and micropores. Specifically, the mesopores in the range of 2–50 nm accounted for 99.83% and 99.45% of the total pore volume for GBFS and UCP, respectively. Therefore, mesopores were the dominant pore type in both GBFS and UCP.

The differential pore size distribution (Figure 13b) revealed that both UCP and GBFS exhibited a bimodal pore size distribution, with their most probable pore sizes being very close to each other, both around 3.5 nm. Furthermore, the pore size distributions of both materials were relatively narrow, falling within the range of 1–50 nm.

#### 3.8.3. The Specific Surface Area, Average Pore Diameter, and Total Pore Volume of UCP

The specific surface area, average pore diameter, and total pore volume of GBFS and UCP, determined by the Brunauer–Emmett–Teller (BET) and Barrett–Joyner–Halenda (BJH) models, are summarized in Table 5. As shown, the BET specific surface area of GBFS was only 0.8809 m^2^/g, with a relatively low total pore volume (0.002853 cm^3^/g) and an average pore diameter of approximately 24.26 nm. In contrast, all physical parameters of UCP exhibited a significant enhancement. The BET specific surface area of UCP reached 2.1732 m^2^/g, which was 2.47 times that of GBFS; the total pore volume was 0.009435 cm^3^/g, approximately 3.31 times that of GBFS; and the average pore diameter was slightly larger at 26.38 nm. These marked improvements clearly demonstrated that UCP possesses a more developed pore structure and a broader effective reaction interface.

It is worth noting that the specific surface areas obtained by the BET method were considerably larger than those measured by the Blaine method (GBFS: 0.388 m^2^/g; UCP: 0.618 m^2^/g). The BET method is generally considered more reliable than the Blaine method, as it does not rely on assumptions regarding particle shape or semi-empirical formulas, and it can comprehensively account for the effects of different pore types [61]. In agreement with the foregoing findings, UCP exhibited a higher total pore volume than GBFS while maintaining a comparable average pore diameter.

The reduced particle size, along with the increased specific surface area and pore volume, not only facilitates the superior physical filling effect of UCP, but also provides additional nucleation sites for C−S−H precipitation and a larger reaction interface with CH. Nevertheless, these microstructural features inevitably give rise to a marked increase in the water demand of UCP.

### 3.9. Discussion

#### 3.9.1. Effect of UCP Composition on the Physical and Chemical Reaction Performance of Cement-Based Materials

UCP exhibited a darker color than GBFS, which was primarily attributed to the incorporation of dark-colored SCMs such as FA and steel slag powder into UCP. At a more fundamental level, this color difference was related to the higher contents of C, Fe, and Mn elements in UCP (Table 3 and Figure 7).

UCP also demonstrated better powder flowability than GBFS (Figure 2). This improvement can be mainly ascribed to its optimized particle size distribution, with particle morphology also playing a contributing role. UCP contains a certain proportion of nearly spherical solid particles and hollow cenosphere structures derived from FA (Figure 9). Such spherical morphology can significantly reduce the contact area between particles, thereby providing a “ball-bearing effect” during particle movement. In contrast, GBFS particles are largely irregular in shape, exhibiting flaky, angular, or fragmental morphologies with high surface roughness (Figure 8), which tend to generate greater resistance during rolling.

Compared with GBFS, the CaO content in UCP was significantly lower. The sum of CaO, MgO, and Al_2_O_3_—which are positively correlated with the reactivity of SCMs—was 59.00% in GBFS versus 51.88% in UCP, while the content of SiO_2_, which is negatively correlated with SCM reactivity [39,62], was 26.14% in GBFS and 27.38% in UCP. These compositional differences suggested that, from a chemical perspective, UCP possesses a lower intrinsic reactivity than GBFS.

However, when GBFS and UCP were separately mixed with water at various ratios, the suspended liquid prepared with UCP exhibited a significantly higher alkalinity. This higher alkalinity is primarily attributable to the reaction of steel slag with water, during which active mineral phases such as C_3_S and C_2_S in steel slag hydrate to form CH, in addition to CH produced during the slaking process of steel slag. Moreover, the higher Na content in UCP (Table 3) may also be related to the additional incorporation of alkaline activators. Given the elevated levels of C and SO_3_ in UCP, it is possible that the sodium-containing alkaline species are primarily present as sodium carbonate and sodium sulfate. The higher alkalinity of UCP after mixing with water is believed to accelerate not only the hydration of UCP particles themselves but also the hydration of cement [63,64], thereby endowing UCP with a higher early-age reactivity.

GBFS was found to exist predominantly as an amorphous phase, with only a minor amount of gypsum. In contrast, UCP contained a relatively small proportion of amorphous phase but a much higher content of crystalline phases. These included inert phases at ambient temperature, such as quartz, mullite, calcium carbonate, and RO phase; reactive phases, including C_3_S and C_2_S; activator phases, such as gypsum, CH, and alkaline activators; as well as superplasticizer. Consequently, the reaction of UCP in cement-based materials is far more complex than that of GBFS.

According to the elemental mapping results, C was the sixth most abundant element in GBFS, following Ca, Si, Al, Mg, and S. Part of this C signal was attributable to the conductive adhesive used during the mapping measurement, while the remainder was mainly derived from raw materials in the ironmaking process—including coke, iron ore, and limestone—as well as from carbonation of GBFS during storage, which forms calcium carbonate. Thus, C in GBFS predominantly exists in the form of carbonates. Due to its relatively low content, it exerts only a minor influence on the performance of cement-based concrete.

In contrast, the C content in UCP was markedly higher, as evidenced by the mass loss attributed to carbonate decomposition in the TG/DTG curves (Figure 5), which increased directly from 0.22% in GBFS to 0.88% in UCP. This elevated C content in UCP originates from multiple sources: in addition to a small amount of unburned C, it includes carbonates from GBFS, calcium carbonate formed by carbonation of hydrated silicate phases in steel slag, added limestone powder, and carbonate-based activators such as sodium carbonate. Calcium carbonate primarily acts as a filler; additionally, it can react with C_3_A in cement clinker to form calcium carboaluminate, which helps stabilize ettringite [58]. Sodium carbonate is a common activator in cement-based materials: it reacts rapidly with calcium hydroxide to form calcium carbonate, thereby accelerating the hydration of cement clinker minerals [65], while the sodium ions provide an alkaline environment that facilitates the dissolution and reaction of SCMs.

GBFS was mainly composed of irregular particles and layered gypsum. In comparison, UCP exhibited a much richer variety of particle morphologies, consisting predominantly of irregular blocky (Particle 8 in Figure 9c), spherical (Particle 3 in Figure 9a), lotus-root-like (Particle 5 in Figure 9a), and porous (Particle 7 in Figure 9c) particles. These diverse shapes endow UCP with higher packing density and improved flowability, while also contributing to its greater adsorptivity.

Although the average particle sizes of GBFS (14.0 μm) and UCP (10.8 μm) were not substantially different, the specific surface area of UCP was far higher than that of GBFS. Specifically, the Blaine specific surface area of UCP was 1.59 times that of GBFS, while its BET specific surface area reached 2.47 times. The finer particle size endows UCP with superior filling ability and a greater number of nucleation sites; however, the larger specific surface area also inevitably increases the water demand of cementitious materials containing UCP.

Both GBFS and UCP were identified as mesoporous materials. Although their average pore diameters and most probable pore sizes were comparable, the total pore volume of UCP was 3.31 times that of GBFS. These pores are mainly derived from the numerous physical defects and microcracks induced during the grinding process, as well as from the porous structure of fragmented FA particles. Such a well-developed pore network undoubtedly enhances the water adsorption capacity of UCP.

#### 3.9.2. Mechanism of Action of UCP in Cement-Based Materials

In summary, the mechanisms by which UCP functions in cement and concrete can be summarized as follows:

(1) Water adsorption. During the ready-mixed concrete stage, the porous structure of UCP adsorbs free water from the cement paste, and the wetting of such a large specific surface area inherently requires additional water. Therefore, the incorporation of UCP undoubtedly increases the water demand of concrete.
(2) Water reduction. The ball-bearing effect of spherical particles in UCP improves the fluidity of the cement paste, thereby reducing the water demand of concrete. Moreover, the addition of superplasticizer to UCP further contributes to water reduction. Consequently, the net effect of UCP on the final workability of concrete is determined by the balance between its water-adsorption and water-reduction effects.
(3) Chemical activation. The alkalis, primarily sodium ions, present in UCP accelerate the early-age hydration of cement. In addition, carbonates and sulfates also promote the early hydration of cement-based materials. Meanwhile, the gypsum in UCP serves to activate the reactivity of both GBFS and steel slag powder.
(4) Filling effect. The extremely fine particle size of UCP enables it to effectively fill the voids between cement particles and the micro-pores within the interfacial transition zone (ITZ), thereby significantly reducing the overall porosity of concrete and refining its pore size distribution. Meanwhile, the abundant ultrafine particles provide numerous heterogeneous nucleation sites for the early precipitation of C−S−H gels, which accelerates the cement hydration process and facilitates the formation of a continuous and uniformly distributed C−S−H gels network. Furthermore, similar to conventional SCMs, the replacement of cement by UCP not only provides more water for the early hydration of cement, but also increases the interparticle spacing of cement grains due to the effective separation by UCP particles, thereby offering greater space for the growth of hydration products such as C−S−H gels.
(5) Pozzolanic reaction. The abundant SiO_2_ and Al_2_O_3_ in UCP react with CH—a hydration product of cement—to form secondary hydration products, including C−S−H gels and calcium aluminate hydrates (C−A−H), which contribute to the improvement of both the later-age strength and durability of cement concrete.

#### 3.9.3. Limitations of the Application of UCP in Cement-Based Materials

Although UCP exhibits a relatively high early-age reactivity and is comparable to GBFS in both performance and cost, certain limitations still remain in its practical application, as outlined below:

(1) Alkali–silica reaction and shrinkage. In the short term, UCP was found to possess a lower amorphous phase content than GBFS, yet it demonstrated higher early-age reactivity. This is mainly attributed to the filling effect of its fine particles and the activating roles of alkalis and gypsum present in UCP. However, the relatively high alkali content raises several concerns. On one hand, the potential risk of alkali–silica reaction (ASR) should be taken into consideration [66]. On the other hand, attention should be paid to whether concrete elements incorporating UCP are more prone to efflorescence. Furthermore, it remains to be determined whether the increased alkali content would restrict the applicable scenarios of UCP—for instance, in steam-curing or alkali-activated environments. Additionally, in field applications, the possible need for extended wet curing should be carefully evaluated, as this may significantly influence the shrinkage and cracking behavior of concrete.
(2) Durability. UCP contains steel slag powder and gypsum, both of which may potentially compromise the soundness of concrete; therefore, their compatibility should be verified prior to use. Steel slag powder contains free CaO (*f*-CaO) and free MgO (*f*-MgO), which are responsible for poor soundness. These two components hydrate slowly and, upon contact with water, react to form calcium hydroxide and magnesium hydroxide, respectively, accompanied by volume expansion that can easily lead to cracking of the hardened concrete. However, ultrafine grinding is considered to accelerate the reaction of *f*-CaO and *f*-MgO, thereby mitigating the soundness issues they would otherwise cause. Whether the steel slag powder in UCP actually induces soundness problems therefore depends on both its dosage and the contents of *f*-CaO and *f*-MgO in the steel slag powder. In addition, gypsum introduces a relatively high SO_3_ content. The Chinese national standard GB 175-2023 [30] specifies explicit limits on SO_3_ content in cement: for Ordinary Portland Cement, the SO_3_ content must be less than 3.5%, whereas for Ordinary Portland Cement containing GBFS, the limit is 4.0%. The SO_3_ content of UCP was determined to be as high as 4.65% (Table 3). The elevated SO_3_ and alkali levels in UCP inevitably raise concerns regarding the long-term expansion risk of cement-based materials. Consequently, the UCP dosage and curing regime should be carefully controlled in practical applications. Furthermore, it is essential to assess the potential adverse effects of UCP on concrete volume stability, particularly through delayed ettringite formation (DEF) and alkali-aggregate reaction (AAR).
(3) Compatibility between superplasticizers. Compatibility between the superplasticizer pre-added to UCP and the superplasticizer used in concrete remains a concern. There exists a wide variety of superplasticizers on the market, and the type incorporated during the production of UCP may differ from that adopted by ready-mix concrete plants in actual batching. Whether these two types of superplasticizers would interfere with each other—potentially leading to a counteractive effect on water reduction—should be further evaluated prior to application.
(4) Performance when blended with other SCMs. Although UCP exhibited slightly better early-age performance than GBFS when used alone as a replacement, in actual concrete production, multiple SCMs are often combined. How UCP performs when used in combination with other SCMs remains unclear and warrants further investigation.
(5) Rapid reactivity evaluation method. The currently available rapid methods for evaluating the early-age reactivity of SCMs were found to be inapplicable to UCP. Therefore, further efforts are required to develop appropriate approaches for the reactivity assessment or performance prediction of concrete containing UCP.

## 4. Conclusions

To elucidate the reasons for the widespread application and high reactivity of ultrafine composite powder (UCP), this study first replaced cement with 50% ground granulated blast-furnace slag (GBFS) and 50% UCP, respectively, and comparatively analyzed the effects of UCP on the setting time of cement paste, the fluidity of mortar, and the compressive and flexural strengths of mortar at 3 d and 28 d. Subsequently, XRF, XRD, TG/DTG, FTIR, elemental mapping, SEM−EDS, and BET techniques were employed to comparatively analyze the appearance, composition, particle morphology, particle size, and pore structure of GBFS and UCP. The results showed that:

(1) At a replacement level of 50%, the setting time of the UCP-blended cement was shorter than that of the GBFS-blended counterpart, while the fluidity of the mortar remained essentially comparable. The UCP-blended mortar exhibited superior compressive and flexural strengths at both early (3 d) and later (28 d) ages. However, the strength growth rate of the UCP-blended mortar between 3 d and 28 d was significantly lower than that of the GBFS-blended mortar.
(2) UCP appeared as a dark gray powder. Its chemical composition showed that the sum of CaO, MgO, and Al_2_O_3_ was considerably lower than that of GBFS, while the contents of C and Fe were higher. After being dispersed in water, the pH value of the UCP suspension was significantly higher than that of the GBFS suspension. Moreover, the mass loss of UCP at 1000 °C was recorded as 5.48%, which was markedly higher than that of GBFS at 1.48%.
(3) UCP was found to be mainly composed of GBFS, FA, steel slag powder, limestone powder, gypsum, superplasticizer, and alkaline activator.
(4) UCP was identified as a porous material, with its pores mainly originating from fragmented porous FA particles, unburned C particles, as well as cracks and defects generated during the grinding process. The pores in UCP were predominantly mesoporous, with mesopore volume accounting for 99.45% of the total pore volume. The BET specific surface area of UCP was 2.47 times that of GBFS, while its Blaine specific surface area was 1.59 times greater; the total pore volume of UCP was 3.31 times that of GBFS, yet the average pore diameter remained comparable between the two materials.
(5) The mechanisms by which UCP functions in concrete include water adsorption, water reduction, chemical activation, filling effect, and pozzolanic reaction. The relatively high early-age reactivity of UCP was primarily attributed to its filling effect, as the fine particles provide additional nucleation sites for cement hydration products, as well as to the chemical activating roles of alkalis and gypsum present in UCP.
(6) In the application of UCP, special attention should be given to its elevated alkali and SO_3_ contents, which may adversely affect the volume stability and durability of concrete. Meanwhile, the compatibility of UCP with various SCMs in composite systems, as well as its adaptability with superplasticizers, also warrants further investigation.

## Figures and Tables

**Table 1:** Key experimental design factors or operating parameters of test methods in this study.

Test Methods	Key Experimental Design Factors or Operating Parameters
Mechanical properties	Mortar specimens of 40 mm × 40 mm × 160 mm were used
The pH measurement	At solid-to-liquid ratios of 1:2, 1:5, 1:8, and 1:10
Setting time	At a water-to-binder ratio of 0.3
Blaine specific surface areas	The sample mass was calculated according to the equation m = ρ × V × (1 − ε), where ρ is the density of the sample, V is the volume of the material bed, and ε is the void fraction.
Particle size distribution	The samples were dispersed using anhydrous ethanol
XRD	At a scan rate of 3°/min
SEM-EDS and elemental mapping	The accelerating voltage was 15 KV
TG/DTG	Under a N_2_ atmosphere at a heating rate of 10 °C/min
FT-IR	At a resolution of 4 cm^−1^
N_2_ adsorption–desorption isotherms	At 77 K over a relative pressure (P/P_0_) range from 0.05 to 0.995.

**Table 2:** Setting time and mortar fluidity of G50 and U50.

Samples	Setting Time (h:min)		Fluidity (mm)
	Initial	Final	
Ref	2:35	3:50	205
G50	5:20	7:10	220
U50	4:55	6:50	215

**Table 3:** The chemical compositions of GBFS and UCP, wt.%.

Materials	CaO	SiO_2_	Al_2_O_3_	MgO	SO_3_	Fe_2_O_3_	MnO	Na_2_O	TiO_2_
GBFS	39.47	26.14	12.34	7.19	3.06	0.39	0.24	0.48	0.67
UCP	31.27	27.38	15.23	5.38	4.65	5.65	1.13	0.85	0.75

**Table 4:** Particle size distribution and Blaine specific surface area of GBFS and UCP.

Sample	Particle Size Distribution (μm)						Specific Surface Area (m^2^/kg)
	D10	D50	D90	Maximum Particle Size	Average Particle Size	Most Probable Particle Size	
GBFS	1.1	10.6	29.4	50.2	14.0	17.8	388.0
UCP	1.0	7.9	23.1	44.8	10.8	14.2	618.0

**Table 5:** N_2_ adsorption analysis of GBFS and UCP.

Sample	N_2_-Specific Surface Area (m^2^/g)	Average Pores Width (nm)	Cumulative Pore Volume (cm^3^/g)
GBFS	0.8809	24.2624	0.002853
UCP	2.1732	26.3784	0.009435

## Data Availability

The original contributions presented in this study are included in the article. Further inquiries can be directed to the corresponding author.

## Abbreviations

The following abbreviations are used in this manuscript:

OPC	Ordinary Portland Cement
SCMs	supplementary cementitious materials
GBFS	ground granulated blast-furnace slag
UCP	ultrafine composite powder
FA	fly ash
ITZ	interfacial transition zone
C_3_S	tricalcium silicate
C_2_S	dicalcium silicate
C−S−H	calcium silicate hydrate
C−A−H	calcium aluminate hydrates
CH	calcium hydroxide
AFt	ettringite
XRF	X-ray fluorescence
XRD	X-ray diffractometer
SEM−EDS	Scanning electron microscope coupled with an X-ray energy dispersive spectrometer
TG−DTG	thermogravimetric and derivative thermogravimetric analyses
FTIR	Fourier transform infrared spectroscopy
BET	Brunauer–Emmett–Teller (BET) analysis
BJH	Barrett–Joyner–Halenda
ASR	alkali–silica reaction
DEF	delayed ettringite formation
